# Arterial pressure and cerebral blood flow variability: friend or foe? A review

**DOI:** 10.3389/fphys.2014.00120

**Published:** 2014-04-07

**Authors:** Caroline A. Rickards, Yu-Chieh Tzeng

**Affiliations:** ^1^Department of Integrative Physiology, Cardiovascular Research Institute, University of North Texas Health Science CenterFort Worth, TX, USA; ^2^Cardiovascular Systems Laboratory, Centre for Translational Physiology, University of OtagoWellington, New Zealand

**Keywords:** blood pressure variability, cerebral blood flow variability, cerebral blood flow (CBF), end organ damage, hemodynamic oscillations

## Abstract

Variability in arterial pressure and cerebral blood flow has traditionally been interpreted as a marker of cardiovascular decompensation, and has been associated with negative clinical outcomes across varying time scales, from impending orthostatic syncope to an increased risk of stroke. Emerging evidence, however, suggests that increased hemodynamic variability may, in fact, be protective in the face of acute challenges to perfusion, including significant central hypovolemia and hypotension (including hemorrhage), and during cardiac bypass surgery. This review presents the dichotomous views on the role of hemodynamic variability on clinical outcome, including the physiological mechanisms underlying these patterns, and the potential impact of increased and decreased variability on cerebral perfusion and oxygenation. We suggest that reconciliation of these two apparently discrepant views may lie in the time scale of hemodynamic variability; short time scale variability appears to be cerebroprotective, while mid to longer term fluctuations are associated with primary and secondary end-organ dysfunction.

## Introduction

Traditionally, clinicians have assessed the cardiovascular status of their patients with static “snapshot” techniques, such as radial pulse for heart rate, brachial sphygmomanometry for arterial pressure, and chest excursions for respiration rate. Subsequently, clinical judgment about health status and identification of potential risk factors was based on average values, without consideration of the inherent dynamic nature of these variables. While the notion that arterial blood pressure is not constant, but fluctuates dynamically over time has been known since the 18th century, the clinical importance of this phenomenon is only now being recognized. There is growing recognition that assessment of hemodynamic variability (e.g., heart rate and arterial pressure) across multiple time scales may provide important insight into acute and long-term clinical outcomes, such as risk of stroke (Shimbo et al., 2012), myocardial infarction (Kjellgren and Gomes, 1993), and end organ damage from hypertension (Mancia et al., 2007; Verdecchia et al., 2007; Leoncini et al., 2013). With advances both in monitoring technologies and data analysis capabilities, we now have the capacity to capture dynamic changes in patient status by recording and analyzing non-invasive, high frequency hemodynamic waveform data, including ECG, arterial pressure, and most recently, cerebral blood flow and oxygenation. These high fidelity recordings have advanced assessment of hemodynamic variability from intermittent day-to-day or visit-to-visit measures, to a beat-to-beat time scale.

In particular, the advent of transcranial Doppler (TCD) ultrasound monitoring in the 1980s by Aaslid et al. (1982) rapidly moved assessment of cerebral blood flow from invasive, technically cumbersome techniques with poor spatial (global cerebral perfusion) and temporal (minutes) resolution [e.g., the Kety-Schmidt diffusible tracer method (Kety and Schmidt, 1948)], to a relatively easy, non-invasive method, overcoming these resolution limitations (i.e., spatial resolution: individual intracranial vessels; temporal resolution: beat-to-beat). TCD ultrasound is now routinely utilized in both the research and clinical settings (Newell and Aaslid, 1992; Willie et al., 2011), and, in combination with non-invasive cerebral oxygen monitoring technologies (e.g., near infra-red spectroscopy, NIRS), continuous assessment of both cerebral blood flow *and* oxygenation are possible.

The evolving appreciation that measurement of hemodynamic variability provides important physiological insight is demonstrated by Newell et al. in the early 1990s who cautioned that the variability of cerebral blood velocity obtained from TCD may “interfere” with measurement of mean values (Newell et al., 1992). Since then, cerebral blood flow variability has been extensively examined in the research setting in an effort to understand underlying regulatory mechanisms, particularly the role of adrenergic (Zhang et al., 2002; Ogoh, 2008; Hamner et al., 2010; Peebles et al., 2012; Purkayastha et al., 2013), cholinergic (Hamner et al., 2012), and myogenic modulation (Langager et al., 2007; Tzeng et al., 2011; Tan et al., 2013) of the cerebral vasculature with changes in arterial pressure. Assessment of cerebral blood flow variability in the clinical setting, however, has lagged the abundance of studies investigating the role of blood pressure variability (BPV), despite clear implications for the subsequent integrity of cerebral tissues (Tzeng et al., 2012b).

Furthermore, the potential role of increased variability in arterial pressure and cerebral blood flow on clinical outcome is somewhat disparate, with studies suggesting both protective (e.g. Sanderson et al., 1972; Allen et al., 2012; Koning et al., 2012), and detrimental (e.g., Lipsitz et al., 1997; Zhang et al., 1998b; Parati, 2005; Kilpatrick et al., 2010; Ko et al., 2010; Rothwell, 2010) effects. This review will provide a brief background on some of the underlying physiological mechanisms responsible for variability in pressure and flow, and then examine the evidence on both sides of the debate, including potential reasons for this apparent dichotomy.

## Time scale of pressure and flow variability

**Figure 1:**
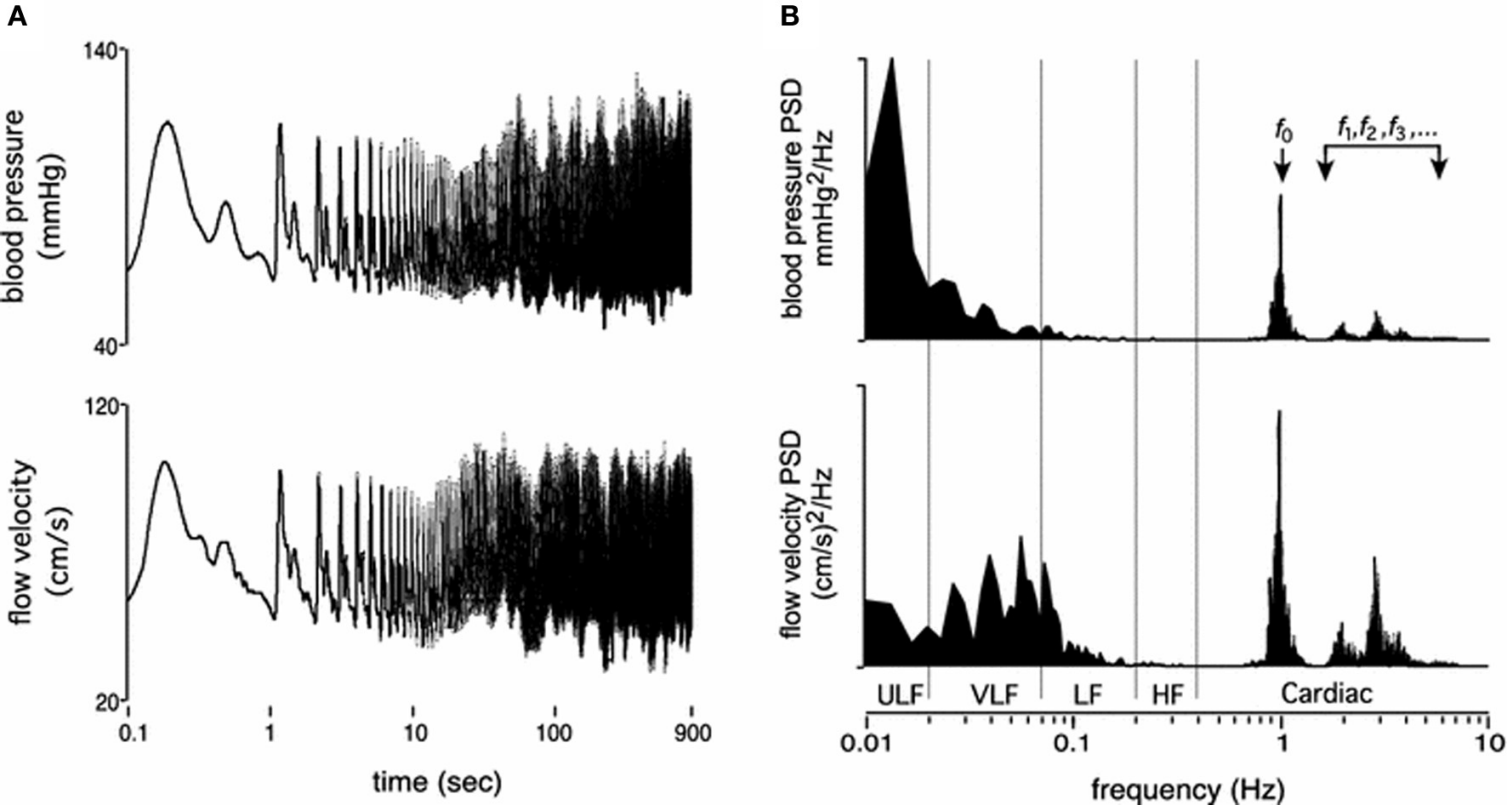
**Resting time domain arterial pressure and cerebral blood velocity tracings (A) and corresponding power spectrums across the frequency range of 0.01–10 Hz (B)**. Panels are presented on log axes. ULF, ultra low frequency; VLF, very low frequency; LF, low frequency; HF, high frequency. f_0_, f_1…_f_n_, refers to the fundamental cardiac frequency and its higher frequency harmonics (From Tzeng and Ainslie, 2014).

Fluctuations in arterial pressure and cerebral blood flow occur across multiple time scales, from beat-to-beat (Figure 1) to day-to-day variability, all associated with a multitude of over-lapping and interacting physiological processes. The most commonly reported metrics of pressure and flow variability include time domain means and standard deviations, which can represent both “static,” long-term variations (hours to days) and short-term (minute to minute) variability, while methods such as power spectral analysis, often assessed using various discrete frequency bands, are generally only suitable for assessment of short-term variations (e.g., 5–20 min).

The underlying physiological mechanisms associated with these different methodological approaches align with the time scale of measurement. For example, changes in mean and standard deviations of pressure and flow over hours to days are most likely associated with long time scale cycles, such as circadian rhythms, hormonal fluctuations, hydration status, fatigue, and associated variations in vascular properties such as compliance (Kotsis et al., 2011; Schillaci et al., 2012; Garcia-Garcia et al., 2013). In comparison, beat-to-beat or minute to minute variability quantifies physiological mechanisms operating within this time scale, such as the cardiac cycle (e.g., approx. ≥1 Hz), respiratory frequency (e.g., high frequency (HF); 0.15–0.4 Hz) (Brown et al., 1993; Cooke et al., 1998), sympathetic/baroreflex modulation (e.g., low frequency (LF); 0.04–0.15 Hz) (Julien, 2006; Stauss, 2007), and myogenic activity (e.g., very low frequency (VLF); 0.004-0.04 Hz) (Stauss, 2007; Tzeng et al., 2011; Tan et al., 2013).

It is important to note that the terminology used to describe hemodynamic variability differs between disciplines; while in the clinical setting, “short-term” variability may describe changes in arterial pressure every 15 min over a 24-h period (Schillaci et al., 2012), this same designation can also be used to describe beat-to-beat variations quantified in the frequency domain. In the case of this review, we will use the latter definition for short-term variability (i.e., 5–10 min recordings with quantification of VLF, LF and HF power), and longer time scales will be described as mid- (within 24-h) and long-term variability (day-to-day, or visit-to-visit).

## “Foe”

The idea that exaggeration of hemodynamic variability may be detrimental for vital organs such as the brain is both physiologically plausible and intuitive. Because the brain has a high metabolic demand for oxygen, any process that enhances perfusion variability has the potential to destabilize tissue oxygenation leading to ischemic injury. Conversely, excessive perfusion can result in the breakdown of the blood-brain barrier, permit the transudation of fluid into the interstitium, and incite hyperperfusion syndromes that are characterized by debilitating neurological sequelae including seizures, headaches, encephalopathy and stroke (van Mook et al., 2005). Therefore, stringent control of cerebral blood flow is pivotal for normal brain function.

However, while it is generally recognized that blood pressure is an important determinant of cerebral blood flow, the exact relationship between pressure and flow is more complex when viewed in light of a contemporary model of cerebral autoregulation (CA) (Tzeng and Ainslie, 2014). The classic model of CA is that cerebral blood flow is maintained constant across a wide range of cerebral perfusion pressures (60–150 mm Hg) (Lassen, 1959). This concept implies that mechanisms normally involved in systemic blood pressure control are relatively unimportant for cerebral blood flow in the presence of intact CA, which is assumed to effect near-perfect compensation of blood pressure variations within the autoregulatory range. Blood pressure, however, is not a static entity that can be described purely in steady-state terms. Blood pressure is highly dynamic and the capacity of the cerebrovasculature to counter-regulate against blood pressure changes is relative and depends on the timescale of the presenting stimuli. Available data supports the idea that the slower, low frequency, components of blood pressure are more effectively buffered than faster, higher frequency, components (such as those associated with respiration), and elevations in blood pressure may be better buffered than reductions in blood pressure (Zhang et al., 1998a). Although the precise mechanisms underpinning these features of CA are still under investigation (Tzeng et al., 2011, 2012a), it is clear that cerebral blood flow exhibits variability, and that BPV is an important determinant of this variability (Tzeng and Macrae, 2013).

Unlike the case for cerebral perfusion variability being a “friend” with protective properties, little data exists that directly implicate perfusion variability being a “foe” for organ function. Rather, the case for exaggerated haemodynamic variability being a negative predictor for organ dysfunction is mainly built on studies of blood pressure dynamics that came initially with the advent of ambulatory blood pressure monitoring in the 1960s (Parati et al., 2001), and since then, other non-invasive blood pressure monitoring devices that allow detailed blood pressure assessment down to timescales of seconds. The successful application of non-invasive blood pressure monitoring technology has firmly established the idea that identifying elevated BPV across a wide range of timescales (as distinct from average blood pressure) may be useful in predicting poorer health outcome (Rothwell, 2010). Notwithstanding the limitations of drawing inferences on cerebral perfusion from blood pressure, we herein present a summary of research that suggest exaggerated systemic hemodynamic variability is associated with poor clinical outcomes.

### BPV and primary organ injury

As previously described, short-term variations in blood pressure are commonly characterized in the frequency domain using power spectral analysis typically of short recordings (e.g. range 5–10 min) (Tzeng and Macrae, 2013). Such variations encompass beat-to-beat changes due to mechanically induced changes in cardiac output caused by respiratory activity (Sin et al., 2010), and longer term fluctuations related to a myriad of cardiovascular control mechanisms including the arterial baroreflex (Tzeng et al., 2009), the renin-angiotensin system (Gouedard et al., 1996), the vascular myogenic response (Bayliss, 1902), and endothelial nitric oxide release (Nafz et al., 1996). As a result, enhanced short term variability may occur due to altered central autonomic drive, impaired arterial baroreflex function, changes in humoral factors, and well as changes in ventilatory parameters (Stauss, 2007). Compared to longer-term variations, which are clearly associated with end-organ disease (Sega et al., 2002), there is little direct evidence implicating short-term BPV in these pathophysiological processes. However, some investigations have suggested a role of increased LF variability in arterial pressure (Lipsitz et al., 1997) and cerebral blood flow (Zhang et al., 1998b) in acute orthostatic intolerance, associated with enhanced vasomotor activity mediated by variations in sympathetic activity (Lipsitz et al., 1997) and impaired CA (reducing the ability to buffer fluctuations in arterial pressure) (Zhang et al., 1998b). Augmentation of spontaneously occurring oscillations in blood pressure and cerebral blood velocity and cerebral oxygenation in the 0.06–0.40 Hz range have also been documented in hypertensive patients (Li et al., 2013). The clinical significance of these hemodynamic changes are presently unclear but may reflect alterations in metabolic and or vascular myogenic function in hypertensive individuals.

Mid-term blood pressure variations are usually defined as blood pressure fluctuations that occur within a 24-h period. There is considerable overlap in the mechanisms that are responsible for both mid-term and short-term blood pressure variations. Therefore, impairment of baroreflex function, or central sympathetic drive can both augment mid-term BPV. In an early investigation into the link between elevated mid-term BPV to end organ damage, Parati et al., showed that 24-h mean blood pressure and 24-h BPV were independently associated with the magnitude of mid-term BPV (Parati et al., 1987). Subsequently, elevated mid-term BPV has been found to be independently linked to a number of outcome measures that indicated widespread vascular and end-organ damage to the heart, blood vessels, and the kidneys. In particular, there is substantive data pointing to an enhanced propensity for carotid intimal media thickening and atherosclerosis progression (Mancia et al., 2001), increased arterial stiffness (Schillaci et al., 2012), and the development of left ventricular hypertrophy (Schutte et al., 2011). Interestingly, the relationships between BPV and left ventricular hypertrophy is apparent in patients with and without elevations in absolute blood pressure (Palatini et al., 1992), suggesting that surveillance for elevated mid-term BPV may help identify apparently normotensive individuals who are otherwise at increased risk of cardiovascular complications. In other studies that have primarily focused on clinical outcome measures, elevated mid-term BPV is associated with a higher risk of coronary artery restenosis after percutaneous coronary intervention (Cay et al., 2011), cognitive dysfunction in the elderly (Sakakura et al., 2007), and increased radiological presence of cerebral micro-bleeds and white matter hyper-intensities on MRI (Liu et al., 2012).

There is also an emerging body of literature relating BPV and disease outcomes based on clinical measurements of day-to-day or visit-to-visit brachial blood pressures that provide information on long-term blood pressure variability (Mancia et al., 2012). In studies involving the general population, day-to-day systolic and diastolic BPV have been linked to increased all-cause mortality, cardiac mortality, and stroke-related mortality (Kikuya et al., 2008; Johansson et al., 2012). Likewise in patients with co-morbidities such as diabetes and chronic kidney disease, visit-to-visit systolic BPV has been linked to elevated risk of death, and accelerated deterioration of renal function (McMullan et al., 2013).

It needs to be acknowledged that while the majority of research has linked elevated mid- and long-term BPV with increased risk of end-organ disease, there is some data suggesting that the impact of BPV are no greater than can be explained by mean pressure alone. For example, Schutte et al., have argued that in a large unbiased population sample (*n* = 2944), BPV does not contribute to risk stratification over and beyond mean systolic pressure (Schutte et al., 2012). However, blood pressure recordings in this particular trial involved five consecutive blood pressure recordings taken on only two occasions separated 2–4 weeks apart. Given the complexities of BPV, it seems that such a protocol is unlikely to yield a comprehensive summary of true BPV.

### BPV and secondary organ injury

**Figure 2:**
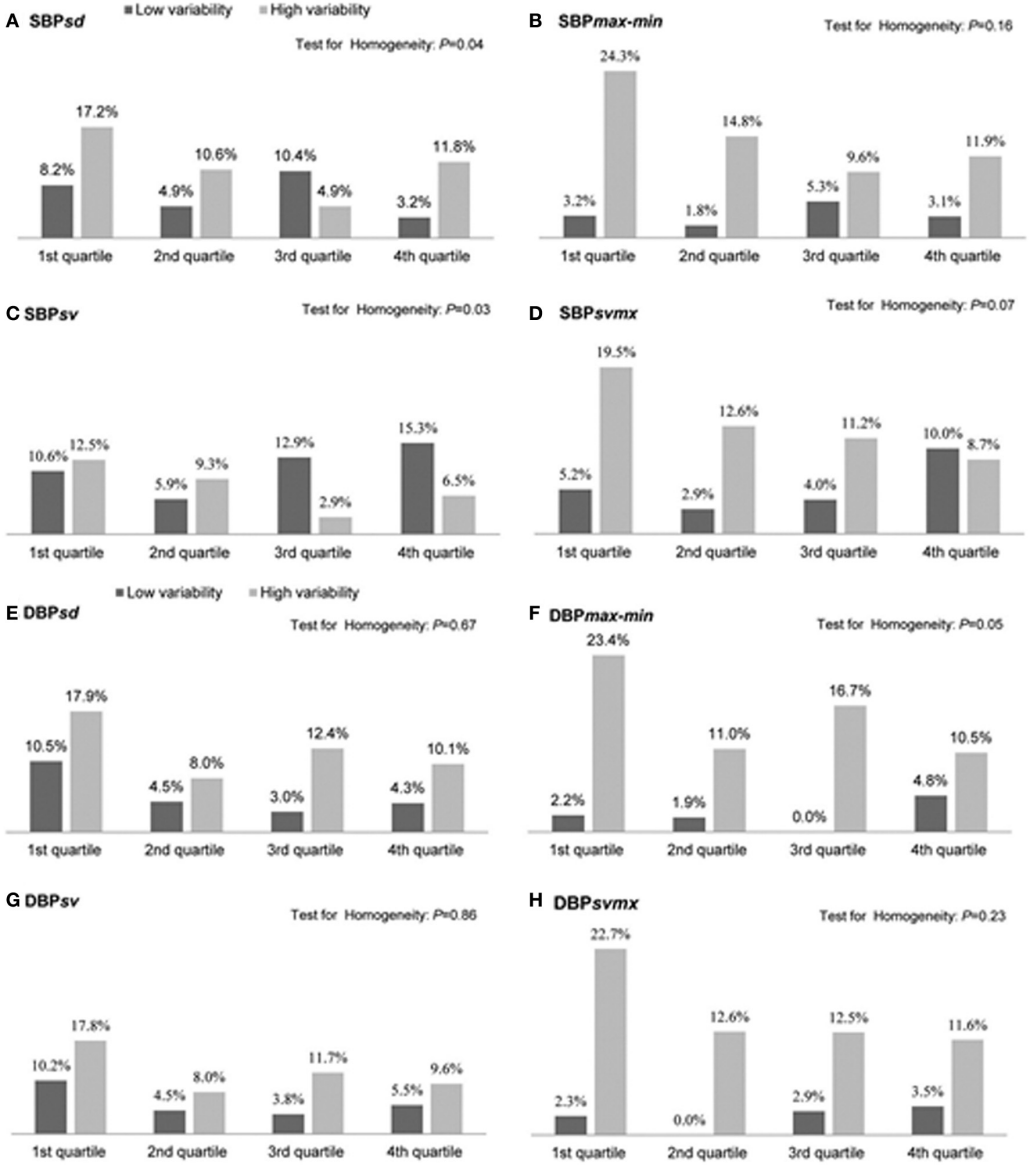
**Proportion (%) of patients with hemorrhagic transformation following an initial ischemic stroke in the low and high blood pressure variability groups of each quartile of mean systolic blood pressure (SBP) or mean diastolic blood pressure (DBP) during the first 72 h**. Measures of blood pressure variability include the standard deviation (SD), difference between maximum and minimum (max-min), the successive variation index (sv), and the maximum successive variation index (svmax). The successive variation was defined as the square root of average difference in blood pressure between successive measurements (From Ko et al., 2010, with permission).

Not only are increases in mid- and long-term BPV associated with accelerated end-organ damage and acute primary cardiovascular events (e.g., acute stroke) (Pringle et al., 2003), there is growing recognition that BPV (beat-to-beat and reading-to-reading) is also a crucial determinant of acute secondary organ damage, once an initial vascular insult has occurred (Stead et al., 2006; Sykora et al., 2009; Tsivgoulis and Ntaios, 2012). Secondary damage is particularly important for organs such as the heart and brain that have no regenerative capacity and have a high metabolic demand for oxygen and therefore low tolerance for hypoxia. Accumulating clinical data shows that patients who are admitted to hospital with acute stroke are more likely to suffer poor function outcomes if BPV is acutely elevated (Dawson et al., 2000). Enhanced beat-to-beat, or reading-to-reading BPV during the acute stroke period predicts the risk of secondary stroke complications such as hemorrhagic transformation of an ischemic stroke (Ko et al., 2010). Because major cardiovascular events such as stroke are often accompanied by increased absolute blood pressure, a major challenge for data interpretation is separating the effects of elevated BPV from that of average increases in mean blood pressure (Schutte et al., 2012). Although Ko et al., has convincingly demonstrated that individuals with high reading-to-reading BPV are more likely to develop secondary complications following stroke event regardless of whether absolute blood pressure is high or low (Ko et al., 2010) (Figure 2), not all studies have fully accounted for likely interactions between these two facets of blood pressure. Further, it must be recognized that Ko et al., did not specifically quantify cerebral perfusion variability. The relationships between elevated BPV and perfusion stability in neurocritical care populations remains poorly understood.

### Mechanisms and implications for enhanced BPV and end-organ damage

The precise mechanisms underpinning the regulation of BPV are multi-factorial and involve factors that drive and attenuate blood pressure changes. Behavioral factors (Pickering et al., 1982) such as physical activity, changes in body posture, and sleep, can induce blood pressure changes at varying time scales. Likewise, centrally driven influences (e.g., sympathetic neural outflow) can also enhance BPV (Poletto et al., 2011; Yoshimoto et al., 2011). Recent genome-wide association studies have identified potential genetic markers for enhanced BPV (Xu et al., 2013), which suggests that BPV elevation may be a heritable trait like hypertension. On the other hand, neural blood pressure control mechanisms, in particular the arterial baroreflex, functions to attenuate blood pressure changes (Bristow et al., 1969). Therefore, the balance between factors that favor and oppose blood pressure variations ultimately determines mid- and long-term BPV.

**Figure 3:**
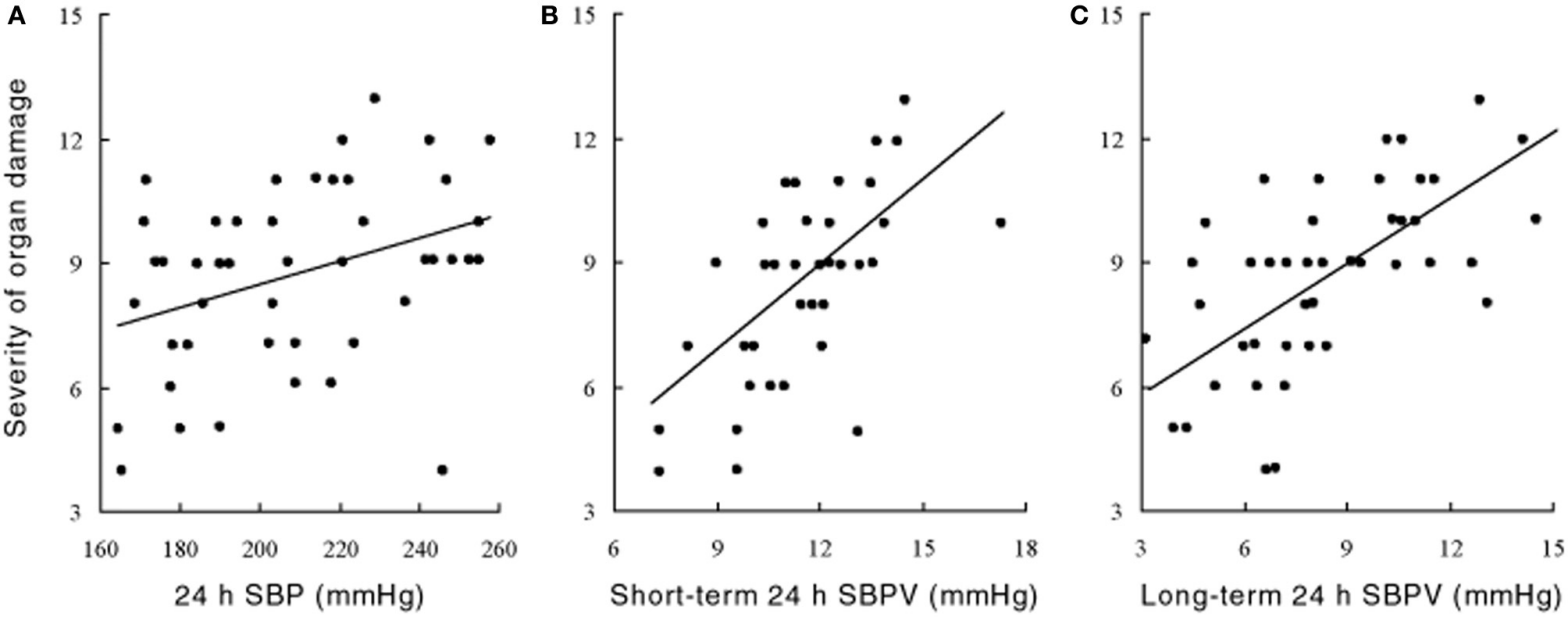
**Relationship between the severity of organ damage and (A) 24 h systolic blood pressure (SBP) and (B,C) its variability (SBPV) in the short (B) and long term (C) spontaneously hypertensive rat at 60 weeks of age**. For **(A)**: *n* = 50, *r* = 0.31, *P* < 0.05; for **(B)**: *n* = 50, *r* = 0.65, *P* < 0.001; for **(C)**: *n* = 50, *r* = 0.63, *P* < 0.001. Severity of organ damage are composite scores calculated from the scoring criteria outlined in Shan et al. (1999). Rats are assigned scores ranging from 0 (no evidence of damage) to 2 (severe damage) for a broad range of clinically relevant gross-anatomic and light micro-scopic end-points. Specific items include left/right ventricular thickness, renal cortical thickness, myocardial infarction/ischaemia, coronary atheroscherosis, thickness of myocardial fibers, atrophy or compensative enlargement of glomeruli, and tubules, Arterial sclerosis and degeneration of kidney, Basilar artery arteriosclerosis of cerebrum, Cerebral hemorrhage or infarction, mesenteric artery hypertrophy, and stroke and hemiplegia (Data and results from Su and Miao, 2001, with permission).

The precise mechanisms underpinning the links between elevated BPV and end-organ damage are not yet fully understood, but available evidence suggest that both functional impairment and structural vascular changes are involved (Figure 3). Several studies have shown in sino-aortic denervated (SAD) rats that a chronic increase in BPV induces aortic hypertrophy and left ventricular hypertrophy (Su and Miao, 2001; Miao and Su, 2002). The mechanical cardiac changes are typified by enhanced cardiac wall thickness and increased total myocardial wall area, and relative reduction in elastin (Su and Miao, 2001). There is also evidence that increased BPV induced by SAD increases cardiac expression of type I and III collagen, and atrial natriuretic peptide (Flues et al., 2012). These findings suggest that the hypertrophic cardiac changes in SAD are the consequence of collagen accumulation in addition to smooth muscle proliferation. The time course of these changes are poorly understood but preliminary evidence suggests that cardiac changes usually arise only after elevations in BPV. In contrast, evidence suggests that vascular changes occur early and can be the first signs of BPV related organ damage. In SAD rats for example, vascular changes emerge as early as 2 weeks whereas cardiac changes occur around 10–16 weeks (Miao and Su, 2002). The trigger for these abnormal structural changes are unclear but enhanced stress on the arterial wall associated with increased rate of blood pressure variations may play a role (Miao et al., 2006). In contrast, the downstream effects of stiff arteries on the cardiovascular system are well recognized. The loss of arterial compliance leads to the loss of the Windkessel function which can lead to enhanced linear transmission pulse waves along the arterial path (Bateman, 2004), as well as increase left ventricular load (Yano et al., 1997). This may explain why vascular changes appear to precede cardiac changes in animal experimental models such as the SAD rat (Miao and Su, 2002). It is important to recognize that associations between BPV and end-organ dysfunction do not imply causality and it is possible that BPV elevation may be the product, rather than the cause of organ damage.

**Figure 4:**
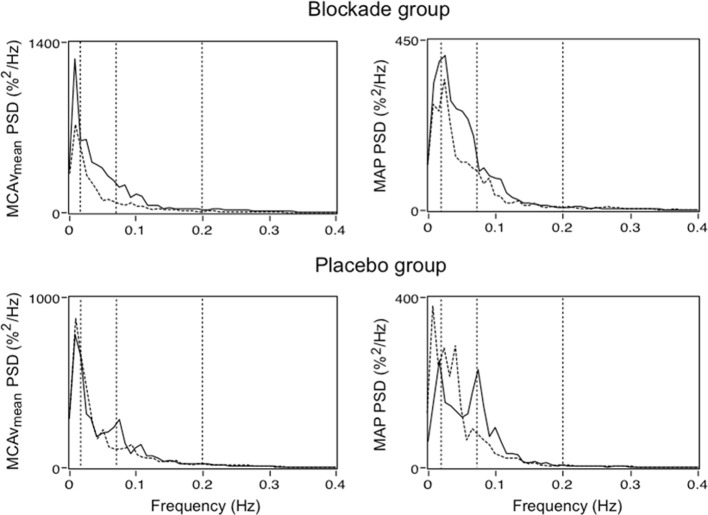
**Power spectral density (PSD) for mean middle cerebral artery flow velocity (MCAv_mean_) and mean arterial blood pressure (MAP) in patients treated with a low dose calcium channel blocker (oral Nimodipine 60 mg) and placebo pill**. Solid and dashed lines represent group median PSD before and following treatment, respectively. Dashed vertical lines illustrate boundaries of the very low frequency (VLF; 0.02–0.07 Hz), low frequency (LF; 0.07–0.20 Hz), and high frequency (HF; 0.20–0.30 Hz) bands (Adapted from Tzeng and Macrae, 2013).

Furthermore, in addition to vascular structural changes, elevated BPV can also lead to end-organ dysfunction by disturbing organ perfusion and oxygenation. Because the vital organs such as the brain and heart have high metabolic demand, any process that enhances perfusion variability has the potential to destabilize tissue oxygenation and therefore result in organ dysfunction. This means that, in addition to BPV, the integrity of flow-stabilizing mechanisms such as CA may partly underlie the relationship between elevated BPV and end-organ disease, particularly in the context of secondary brain injury (Reinhard et al., 2005, 2012; Aries et al., 2010). The complexities that CA introduces may also be of importance when considering the pathogenesis of BPV related disease as well as therapeutic treatment effects. Recently, Matsui et al. (2012) reported that day-by-day BPV is lower in patients treated with an angiotensin II receptor blocker/calcium channel blocker combination compared to those treated with an angiotensin II receptor blocker/diuretic combination. This raised the possibility that elevated BPV and or cerebral blood flow variability can be treated using conventional antihypertensive agents (Parati and Bilo, 2012) (Figure 4).

## “Friend”

While a wealth of studies have assessed the *negative* clinical consequences of high BPV (and most likely variability in cerebral blood flow) as highlighted in the “foe” section of this review, the potentially *protective* effect of pulsatile arterial pressure and/or cerebral blood flow has also been demonstrated in a variety of experimental and clinical settings, focused primarily on what we define as “short-term” variability.

### “High” frequency pulsatile flow

**Figure 5:**
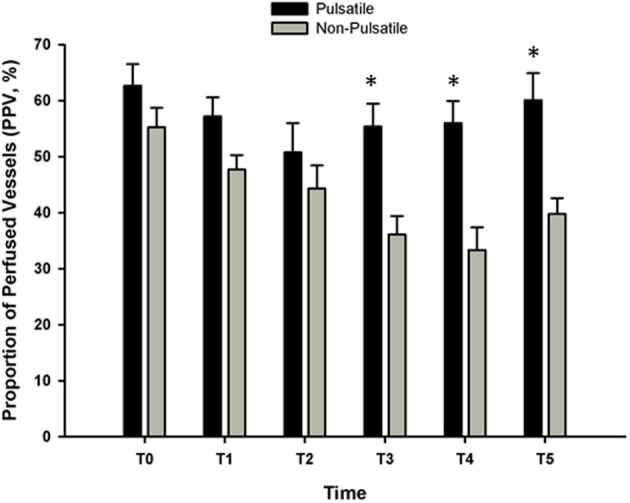
**The proportion of perfused sublingual microvessels (PPV, %) with and without pulsatile perfusion before, during, and after cardiac bypass surgery (CPB)**. Sublingual microcirculation was assessed using orthogonal polarization spectral imaging. Three to five 30 s steady state video images were obtained from the lateral side of the tongue, 2 cm from the tip at each time point. An investigator blind to the study performed the analysis of each video clip. *T*_0_ = baseline; *T*_1_ = 30-min on CPB; *T*_2_ = 90-min on CPB; *T*_3_ = 1 h post CPB; *T*_4_ = 24 h post CPB; *T*_5_ = 48 h post CPB. For the data presented, blood flow was classified as “normal” at approximately 250–350 μm/s. At each time point, the number of vessels that were classified as “normal” was divided by the total number of vessels and reported as PPV%. (Data modified from O'Neil et al., 2012). ^*^P < 0.05 compared with non-pulsatile group.

In 1972, Sanderson et al. (1972) demonstrated that pulsatile cerebral blood flow was associated with decreased neuronal damage following prolonged cardiac arrest in dogs (up to 3 h). The frequency of the pulsatile perfusion was 1.7 Hz (i.e., 100 cycles/minute), consistent with the estimated heart rate, and was able to generate pulse pressures of 25–90 mm Hg, compared with only 2–15 mm Hg with non-pulsatile perfusion (Sanderson et al., 1972). Mean arterial pressure and blood flow, however, were approximately 50 and 12% lower with pulsatile perfusion, but peripheral vascular resistance was also 57% lower; increased shear stress with pulsatile perfusion increases endothelial nitric oxide production (Nakano et al., 2000; Lanzarone et al., 2009), subsequently decreasing resistance (Nakano et al., 2000). The observed decrease in neuronal damage indicates that pulsatile flow improves perfusion and oxygenation of vital tissue, even with lower perfusion pressures. In reviewing the literature, Sanderson et al. (1972) suggest that pulsatile flow may also promote baroreflex-mediated vasodilation, increase the rate of tissue respiration, reduce cerebral critical closing pressure, and prevent the depression of kidney function. In a study of prolonged cardiopulmonary bypass (up to 80 min) combined with hypothermia in dogs, Onoe et al. also demonstrated an increase in cerebral blood flow with pulsatile perfusion, although once rewarming was complete this only persisted in the group arrested for 60 min (Onoe et al., 1994). While some of these findings have been substantiated in subsequent studies of extracorporeal perfusion with cardiac bypass surgery in patient populations, with increased microcirculatory perfusion of the sublingual mucosa (Koning et al., 2012; O'Neil et al., 2012) (Figure 5), increased oxygen consumption (Koning et al., 2012), reduced leukocyte activation (O'Neil et al., 2012), and decreased morbidity and mortality (Taylor et al., 1982; Murkin et al., 1995), pulsatile perfusion therapy is still not a standard procedure in this setting (Hornick and Taylor, 1997; Murkin, 2006; O'Neil et al., 2012).

Pulsatile flow has also been shown to improve cerebral hemodynamic status in other clinically relevant states, including prolonged cerebral ischemia as a model of stroke (Allen et al., 2012), and severe hemorrhage (Bassuk et al., 2010). In a pig model of prolonged (30-min) isolated global normothermic brain ischemia, Allen et al. (Allen et al., 2012) demonstrated that 20-min of pulsatile perfusion at a frequency of 1.3 Hz (80 cycles/min) and a flow rate of 750 ml/min resulted in reduced neurological deficit and cerebral tissue edema, no post-ischemic seizures (compared to 100% of animals in the non-pulsatile perfusion group), and an attenuated increases in oxygen radical damage. While these studies show benefit with pulsatile perfusion at or around the cardiac frequency, Adams et al. (Adams et al., 2000, 2001) have introduced a novel approach to induce pulsatile flow by applying whole body periodic acceleration in the head-to-foot axis (Gz) at a frequency of approximately 3–4 Hz (180–240 cycles/min) and an acceleration of ±0.4 m/s^2^. These investigators have demonstrated that periodic acceleration can increase vital organ blood flow in pigs, including the brain, heart, kidneys, and liver at rest (Adams et al., 2001), and following significant clinical events such as severe hemorrhage (Bassuk et al., 2010), and cardiac arrest (Adams et al., 2011); subsequent survival from hemorrhage also increased from 0 to 50% (Bassuk et al., 2010). In studies using rats and piglets, these protective effects have been shown to be mediated by shear-stress induced release of vasoactive mediators including endothelial nitric oxide, prostacyclin, and prostaglandin E2, with subsequent vasodilation leading to improved tissue perfusion (Adams et al., 2005; Uryash et al., 2009). The potential role of periodic acceleration in recovery from stroke has also been established, with reduced brain damage up to 7 days following ischemia, indicated by reduced infarct size, and decreased markers of autophagy (beclin 1) and apoptosis (fractin) (Martinez-Murillo et al., 2009). Combined, these studies provide intriguing evidence in support of pulsatile perfusion induced by periodic acceleration as a therapy for the protection of cerebral tissues in a variety of clinical scenarios that challenge cerebral blood flow and oxygenation, such as ischemic stroke; human studies investigating these effects appear warranted.

Pulsatile flow patterns around the cardiac frequency can also be assessed via calculation of pulsatility, generally derived as systolic-diastolic/mean flow. Studies have reported a role of increased cerebral blood flow pulsatility and tolerance to central hypovolemia in healthy human subjects following head-up tilt and lower body negative pressure (LBNP) (Thomas et al., 2009), in hemorrhaging sheep (Lewis et al., 1999), and in patients with head injury (Czosnyka et al., 1994). In these studies, increased pulsatility resulted from a reduction in diastolic cerebral blood velocity, and was interpreted as a mechanism of protecting blood supply to the cerebral tissues with decreasing perfusion pressures (Czosnyka et al., 1994; Lewis et al., 1999; Thomas et al., 2009). Less energy may be required to maintain forward flow if the flow is pulsatile vs. non-pulsatile, as higher mean flow rates can be generated for equal mean arterial blood pressures (Shepard et al., 1966; Sanderson et al., 1972; Czosnyka et al., 1994).

### “Low” frequency pulsatile flow

The studies described thus far have utilized pulsatile perfusion therapy at frequencies at or above the cardiac frequency (i.e., ≥1 Hz). Other investigations, however, have also assessed pulsatile flow at much lower frequencies, generally associated with patterns of respiration, sympathetic nerve activity, and myogenic activity, among other factors.

**Figure 6:**
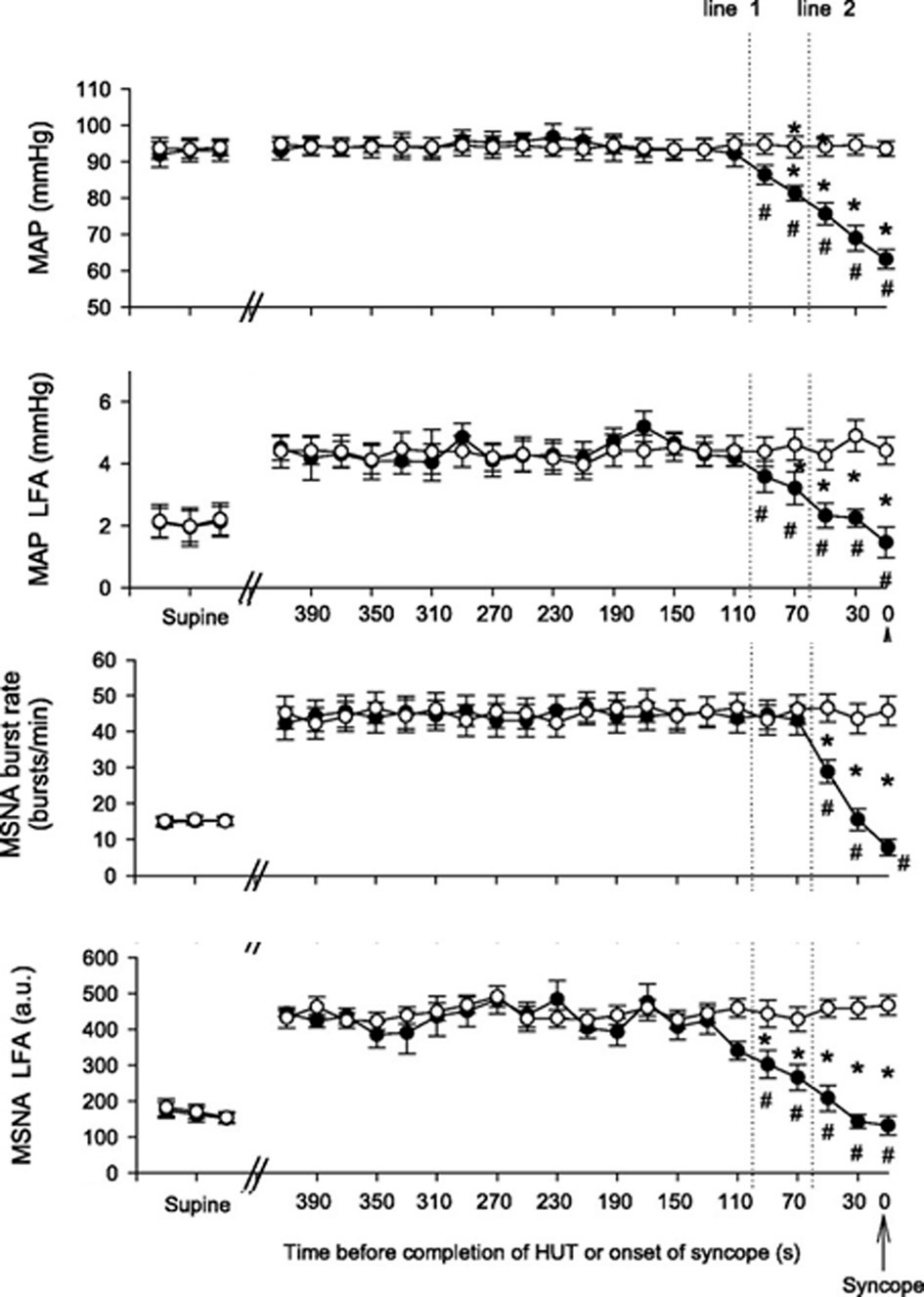
**Mean arterial pressure (MAP), MAP low frequency amplitude (LFA), muscle sympathetic nerve activity (MSNA), and MSNA LFA responses to head-up tilt (HUT) in syncopal (closed circles) and non-syncopal subjects (open circles)**. Line 1 and line 2 represent 100 and 60 s time points prior to presyncope or completion of the HUT protocol (From Kamiya et al., 2005, with permission).

**Figure 7:**
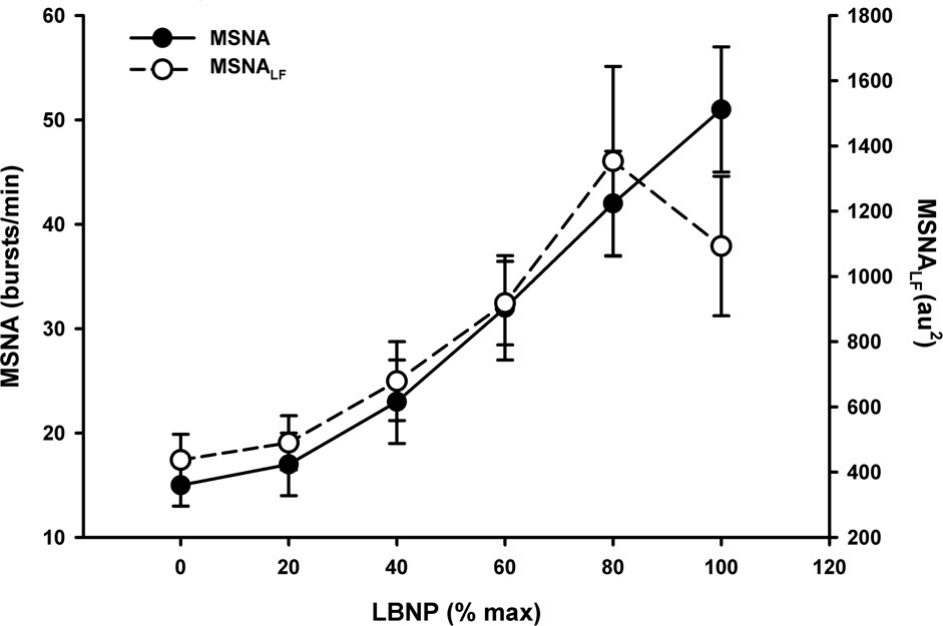
**Muscle sympathetic nerve activity (MSNA) and MSNA low frequency (LF) power in response to progressive central hypovolemia induced via lower body negative pressure (LBNP)**. Data presented as a percentage of maximum LBNP tolerance (Data modified from Cooke et al., 2009).

**Figure 8:**
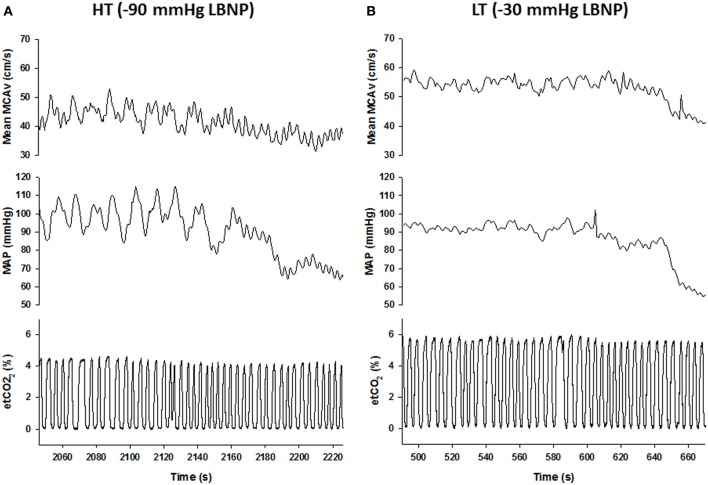
**Mean middle cerebral artery velocity (MCAv), mean arterial pressure (MAP), and end-tidal (et) CO_2_ responses during progressive central hypovolemia in a representative high tolerant (HT; A) and low tolerant (LT; B) subject**. Data are from the final 3-min prior to presyncope. Note the enhanced oscillatory characteristics of the MCAv and MAP tracings in the HT subject compared with the LT subject, and the transition from low frequency (LF) to high frequency (HF) oscillations at presyncope in the HT subject (From Rickards et al., 2011).

**Figure 9:**
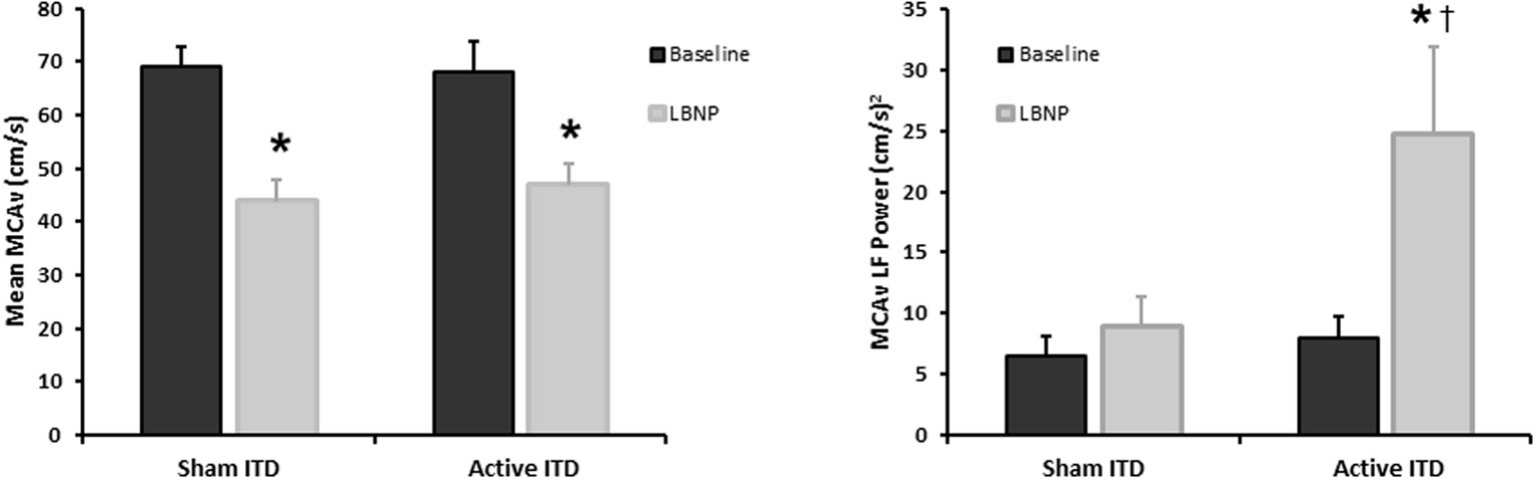
**Mean middle cerebral artery velocity (MCAv), and MCAv low frequency (LF) power during progressive central hypovolemia induced by lower body negative pressure (LBNP) while breathing through a sham or active inspiratory threshold device (ITD)**. Breathing through an ITD further decreases intra-thoracic pressure upon inspiration, subsequently increasing venous return and stroke volume (Data modified from Rickards et al., 2007). ^*^P < 0.05 compared with baseline, ^†^*P* < 0.05 compared with sham condition.

Under conditions of experimentally induced central hypovolemia in healthy human subjects, such as LBNP and head-up tilt, increased oscillatory power in arterial pressure and/or cerebral blood flow has been associated with increased tolerance to these stressors. In a number of studies using head-up tilt alone, or combined with LBNP, individuals with poor tolerance (i.e., display symptoms of presyncope or syncope) exhibited reduced LF oscillations in arterial pressure (Gulli et al., 2001; Kamiya et al., 2005) and muscle sympathetic nerve activity (MSNA) (Kamiya et al., 2005), compared with non-syncopal subjects who showed a persistent elevation in LF power (Figure 6). The reduction in blood pressure and MSNA LF variability was associated with reduced absolute MSNA (Kamiya et al., 2005). Additional studies have demonstrated a clear relationship between changes in the LF oscillatory characteristics of MSNA and the magnitude and direction of MSNA responses, such as during severe hypovolemic stress (Cooke et al., 2009) (Figure 7), underscoring the role of sympathetic variability on arterial pressure variability within the LF range. In studies assessing physiological responses to pre-syncopal limited LBNP, we observed increases in reflex-mediated endogenous LF variability in arterial pressure *and* cerebral blood velocity in subjects with high tolerance (HT) to this stress compared with low tolerant (LT) subjects (Rickards et al., 2011); this was despite similar reductions in absolute arterial pressure and cerebral blood flow between groups (~20–30%) (Figure 8). Breathing through an inspiratory threshold device also increased LF variability in arterial pressure and cerebral blood flow in subjects undergoing LBNP, which was associated with the delayed onset of presyncopal symptoms, and increased tolerance (Rickards et al., 2007). These exogenously-induced oscillations were also coincident with profound reductions in absolute cerebral blood velocity, which was not protected compared with the control condition (i.e., no resistance breathing) (Rickards et al., 2007) (Figure 9). Interestingly, posture dependent increases in LF power of cerebral oxygen (derived from NIRS) have also been shown in healthy subjects transitioning from the supine to seated, or supine to standing position, coincident with higher LF power in arterial pressure. Absolute cerebral oxygen levels were not protected, however, and the role of these oscillations on orthostatic tolerance was not assessed (Tachtsidis et al., 2004).

In these aforementioned studies where respiration was reported, the elevation in LF power was not associated with breathing rate, as subjects, on average, were breathing outside of the LF range (i.e., >0.15 Hz or >9 breaths/min) (Gulli et al., 2001; Kamiya et al., 2005; Rickards et al., 2007, 2011). There is, however, a potentially important role of breathing rate in the generation of LF variability in arterial pressure and cerebral blood flow, which can lead to improved orthostatic tolerance. Recently, Lucas et al. (2013) observed a 15% increase in tolerance to combined head-up tilt and LBNP in subjects breathing at a fixed rate of 6 breaths/min (i.e., 0.1 Hz) vs. spontaneous breathing at 16–20 breaths/min (i.e., 0.27–0.33 Hz), thereby forcing a marked increase in LF power of mean arterial pressure and mean cerebral blood velocity; again, the reduction in cerebral blood velocity was similar between the two breathing conditions, so did not account for the improvement in tolerance.

While these improvements in tolerance to central hypovolemia were not associated with the preservation of absolute cerebral blood flow, the effect of LF pulsatile cerebral blood flow on cerebral tissue oxygenation is a plausible underlying mechanism based on the animal and human clinical studies outlined above using pulsatile perfusion at higher frequencies. Some of these studies have demonstrated the role of nitric oxide-induced vasodilation and improved oxygen delivery at high oscillatory frequencies (Nakano et al., 2000; Lanzarone et al., 2009; Uryash et al., 2009; Adams et al., 2011). Additionally, forcing oscillations in arterial pressure at 0.1 Hz has also been shown to elicit an acute antihypertensive effect (over the first 8 h of a 24 h recording) in a dog model via liberation of nitric oxide (Nafz et al., 2000), which could improve tissue perfusion and oxygenation. The role of LF cerebral blood flow variability on the release of nitric oxide and subsequent regulation of cerebral blood flow in humans, however, is unclear. Zhang et al. showed that inhibition of nitric oxide synthase (NOS; via infusion of L-NMMA) did not alter the generation of LF oscillations in cerebral blood velocity or arterial pressure in healthy humans at rest or during head-up tilt, and did not affect transfer function estimates of cerebral autoregulation (Zhang et al., 2004). Cerebral blood flow at rest and in response to head-up tilt were also not affected by NOS inhibition, suggesting that nitric oxide may not be liberated from the endothelium under conditions of increased LF variability. It is possible, however, that other shear-stress induced vasoactive mediators may be improving perfusion and oxygenation of the cerebral tissues under these conditions, such as histamine (DeForrest and Hollis, 1978), or prostaglandins, but these effects have not been elucidated. In comparison, while cerebral blood flow responses were not assessed, Castellano et al. (1995) demonstrated that NOS inhibition reduced systolic arterial pressure LF power for up to 40-min following infusion of L-LMMA, likely due to baroreflex-mediated reductions in sympathetic activity as a result of increased arterial pressure. These investigators only assessed these effects at rest, however, and did not perturb the system (e.g., forcing 0.1 Hz oscillations, or performing a head-up tilt maneuver to induce sympathetically-mediated LF oscillations) to determine if LF power was also reduced under these conditions.

It has been speculated that the spontaneous generation of oscillations in cerebral blood flow and cerebral oxygenation in the lower frequency ranges (i.e., <1 Hz) is associated with changes in vascular properties, such as arterial compliance (Schroeter et al., 2004, 2005). Theoretically, a decrease in arterial compliance could result in increased oscillatory characteristics of arterial pressure and cerebral blood flow due to the reduced buffering capacity of the vasculature. However, in a study of elderly subjects (62–71 years), LF variability in cerebral oxygenation (via NIRS as a measurement of the microvasculature) was lower at rest and during a visual stimulation task compared with young subjects (19–29 years) (Schroeter et al., 2004). These investigators suggested that this response was due to decreased compliance of the arteries with aging, and a reduction in the reactivity of the microvascular smooth muscle cells (Schroeter et al., 2004), although they did not quantify arterial compliance to confirm this hypothesis. Similarly, in studies of patients with cerebral microangiopathy (Schroeter et al., 2005) or a history of cerebral infarction (Li et al., 2010), conditions associated with an increase in arterial stiffness (and decreased arterial compliance), spontaneous LF (Schroeter et al., 2005; Li et al., 2010) and VLF (Li et al., 2010) power of oxy-hemoglobin was also reduced compared with age matched controls, but again, cerebral vascular compliance was not directly assessed. Finally, VLF BPV was lower in stroke-prone hypertensive rats compared with stroke-resistant hypertensive rats, reflective of reduced cerebrovascular myogenic function, which usually protects the brain from hemorrhagic stroke (Stauss et al., 2008). The direct role of arterial compliance on the generation of these short-term oscillations in arterial pressure, cerebral blood flow, and/or cerebral oxygenation has not been clearly quantified, and should be investigated further.

## Conclusion

In this review we have attempted to highlight the complexities inherent in the characterization of hemodynamic variables such as blood pressure and cerebral blood flow. We have contrasted evidence that supports hemodynamic variability as a protective feature of physiology against evidence suggesting that hemodynamic variability heralds expansive damage to organ function. Our review suggests that reconciliation of these two apparently discrepant views may lie in the time scale of hemodynamic variability; short time scale variability appears to be cerebroprotective, while mid-to-longer term fluctuations are associated with primary and secondary end-organ dysfunction. The extent to which knowledge of the positive and deleterious influences of hemodynamic variability will lead to improve health outcomes are presently unknown, but the case is mounting against classical approaches to hemodynamic assessment that focuses narrowly on absolute blood pressure and/or cerebral blood flow.

## Author contributions

Caroline A. Rickards and Yu-Chieh Tzeng contributed equally to this manuscript in terms of conception of the work, drafting the work and revising it critically for important intellectual content, and final approval of the version to be published. Caroline A. Rickards and Yu-Chieh Tzeng agree to be accountable for all aspects of the work in ensuring that questions related to the accuracy or integrity of any part of the work are appropriately investigated and resolved.

### Conflict of interest statement

The authors declare that the research was conducted in the absence of any commercial or financial relationships that could be construed as a potential conflict of interest.
